# EI24 Suppresses Tumorigenesis in Pancreatic Cancer via Regulating c-Myc

**DOI:** 10.1155/2018/2626545

**Published:** 2018-10-02

**Authors:** Yi Zang, Lei Zhu, Tong Li, Qi Wang, Juanjuan Li, Yuting Qian, Lumin Wei, Mingping Xie, Wen-Hao Tang, Xu Liu, Ying Zhu, Lifu Wang

**Affiliations:** ^1^Department of Gastroenterology, Ruijin Hospital Affiliated to Shanghai Jiao Tong University School of Medicine, Shanghai 200025, China; ^2^Department of Gastroenterology, Changzheng Hospital, Second Military Medical University, Shanghai 200003, China; ^3^Department of General Surgery, Huadong Hospital Affiliated to Fudan University, Shanghai 200040, China; ^4^Department of Surgery, Yancheng No. 1 People's Hospital, Yancheng, Jiangsu 224005, China

## Abstract

The EI24 autophagy-associated transmembrane protein is frequently associated with tumor growth and patient survival. In the present study, we found that EI24 was downregulated in pancreatic ductal adenocarcinoma (PDAC) tissues compared with adjacent normal tissues and was associated with cancer cell differentiation. Overexpression of EI24 suppressed cancer cell growth *in vitro* and *in vivo* and induced cell cycle S phase arrest, with no impact on caspase-dependent apoptosis. EI24 overexpression also resulted in reduced c-Myc expression, an oncogene in PDAC, accompanied with increased LC3B-II formation, increased Beclin-1, and diminished p62. Together, we propose that EI24 suppresses cell proliferation and prompts cell cycle arrest in pancreatic cancer cells by activating the autophagic lysosomal degradation of c-Myc. Our results suggest a potential mechanism underlying the antitumor effects of EI24 in PDAC and provide insight into the crosstalk between autophagy and cell proliferation involving a possible EI24/Beclin-1/p62/c-Myc signaling pathway.

## 1. Introduction

Pancreatic ductal adenocarcinoma (PDAC) is one of the most lethal cancers, with a 5-year survival rate of less than 3% [1]. The poor prognosis of PDAC is attributed to many factors, including late detection, early recurrence and metastasis, and resistance to chemotherapy and radiotherapy. Researchers have revealed genetic characteristics of PDAC [2, 3] and c-Myc, an important transcription factor that is involved in various cellular processes including cell proliferation, differentiation, and apoptosis and acts as an oncogene in PDAC [4]. However, the underlying molecular mechanisms that regulate tumor initiation and progression in PDAC remain poorly understood.

The EI24 (etoposide-induced 2.4 kb transcript) gene is located on human chromosome 11q23, and its low expression has been linked with invasive breast carcinomas and cervical cancers [5, 6]. EI24 functions as a tumor suppressor and plays an important role in the negative regulation of cell growth and induction of apoptosis [5, 7, 8]. In contrast, other studies reported that reduced EI24 expression attenuated DMBATPA-induced skin carcinogenesis, suggesting a potential role for EI24 in tumor promotion [9]. These studies implicate diverse and complex functions of EI24 in tumorigenesis. Our previous microarray analysis showed that EI24 mRNA was upregulated in human PDAC tissues compared with paraneoplastic tissues (2.69-fold increase; *P* = 0.003) [3]. The role of EI24 in pancreatic cancer remains enigmatic.

Autophagy is a cellular mechanism for the clearance and degradation of cellular proteins and organelles. The role of autophagy in cancer cells is complex, with both demonstrating tumor-suppressive and tumor-promoting effects [10]. In PDAC cells, autophagy usually functions as a cytoprotective strategy to cope with stress, while induction of autophagy has also been reported to enhance cell death [10]. Beclin-1 takes part in the initial steps of autophagy. p62 is an autophagic cargo protein and binds to substrates during autophagic lysosomal degradation. The previous report showed that activating Beclin-1-induced p62-mediated autophagic lysosomal degradation of c-Myc resulted in the inhibition of breast cancer cell proliferation [11]. Recent studies have demonstrated that EI24 functions as an autophagy-associated transmembrane protein and is an essential component of basal autophagy in mammals [12]. Furthermore, EI24 plays an important role in the ubiquitin-proteasome system-autophagy crosstalk [13]. Whether the functions of EI24 in autophagy are related with its activities in tumor cells is not yet clear.

In the current study, to clarify the role of EI24 in PDAC, we examined the expression and functions of EI24 in PDAC tissues and cancer cell lines compared with controls and its effects on tumorigenesis *in vivo*. We further examined the induction of autophagy by EI24 in PDAC cells to uncover a potential mechanistic link with its effects on cancer cells.

## 2. Materials and Methods

### 2.1. Patients and Tissue Samples

Thirty-six pairs of PDAC tissues and adjacent normal tissues were collected from Ruijin Hospital affiliated to Shanghai Jiao Tong University School of Medicine. None of the patients received radiotherapy or chemotherapy before surgery. Formalin-fixed and paraffin-embedded tissues were used for further analysis. Histological diagnoses were performed by two senior pathologists independently. Twelve pairs of fresh specimens were quick-frozen in liquid nitrogen after surgery and stored at −80°C. This study was approved by the ethics committee of Ruijin Hospital, and all participants gave informed consent.

### 2.2. Cell Culture

Human pancreatic cancer cell lines CFPAC-1, SW1990, AsPC-1, and PANC-1 were purchased from the Cell Bank of the Chinese Academy of Science (Shanghai, China). CaPan-1 and CaPan-2 were purchased from the American Type Culture Collection and preserved by our laboratory [3]. CaPan-1, CaPan-2, SW1990, and PANC-1 cells were grown in DMEM; CFPAC-1 cells were grown in IMDM; and ASPC1 cells were grown in RPMI 1640. All cell culture media were supplemented with 10% fetal bovine serum, 100 *μ*g/ml penicillin, and 100 *μ*g/ml streptomycin, and cells were cultured at 37°C with 5% CO_2_ in a humidified incubator.

### 2.3. Quantitative Real-Time PCR (qRT-PCR)

Total RNA was extracted from frozen tissues and cells using TRIzol (Invitrogen, Eugene, OR, USA), and cDNA synthesis was performed using the First Strand cDNA Synthesis kit (Yeasen, Shanghai, China) according to the manufacturer's instructions. qRT-PCR analysis was conducted using the SYBR Green Supermix kit (Yeasen) on the StepOnePlus™ Real-Time PCR System (Applied Biosystems, Waltham, Massachusetts, USA) as described in the manufacturer's instructions. Relative mRNA expression levels were calculated using the 2^−ΔΔCT^ relative quantification method using *β*-actin as a control. The primers are shown in Supplementary Table 1.

### 2.4. Western Blot Analysis

Tissues or cells were lysed and centrifuged. Then, the proteins were subjected to SDS-PAGE and transferred onto polyvinylidene fluoride membranes, and the membranes were processed as described previously [3]. Primary antibodies and secondary antibodies are shown in Supplementary Table 2. Immunoreactivity was detected by the Enhanced Chemiluminescence kit (GE Healthcare, Chicago, Illinois, USA) and visualized with the G Box Chemic XL system (Syngene, Cambridge, UK). Relative protein expression was quantified by normalizing against *β*-tubulin using the Quantity One software (Bio-Rad, Hercules, CA, USA).

### 2.5. Immunohistochemistry (IHC)

IHC staining was performed using standard techniques as described previously [3]. Primary antibodies included antibodies against EI24 (1 : 50, Santa Cruz Biotechnology, Dallas, Texas, USA), Ki67 (1 : 200, Servicebio, Wuhan, China), and c-Myc (1 : 100, Santa Cruz Biotechnology). To evaluate EI24 and c-Myc expression in human tissues, we used a semiquantitative method by scoring both the percentage of positive cells and staining intensity as follows: percentage of positive cells (0, 0%; 1, <25%; 2, 25%–50%; 3, 51%–75%; and 4, >75%) and the staining intensity (0, negative; 1, weak staining; 2, intermediate staining; and 3, strong staining). The two scores were then multiplied to determine the final score. We defined the samples having a final score < 4 as low expression and samples with a score of ≥4 as high expression. Each slide was independently evaluated by two senior pathologists.

### 2.6. Plasmid Construction and Stable Transfection

To knockdown EI24 in CaPan-2 cells, the specific antihuman EI24 shRNA sequence (5′-GAGAGTGAGCCACGTATTGTT-3′) was constructed into lentivirus using pGMLV-SC5 (Genomeditech, Shanghai, China). To overexpress EI24 in PANC-1 cells, the lentivirus-mediated Flag-tagged overexpression of human EI24 was constructed using the pHMGV1-EGFP-Puro vector (Humangen, Shanghai, China). The empty vectors were used as negative controls. For lentiviral infection, PANC-1 cells or CaPan-2 cells were transfected in 6-well plates with experimental constructs or the respective controls using polybrene (Genomeditech) following the manufacturer's protocol. To establish stable transfected cells (CaPan-2-shEI, CaPan-2-shCTR, PANC-1-EI, and PANC-1-SCR), cells were grown in culture medium with 10 *μ*g/ml puromycin.

### 2.7. Cell Proliferation Assay

Cell viability was measured using the Cell Counting Kit-8 (Yeasen) according to the manufacturer's protocol. Stable transfected pancreatic cancer cells were plated in 96-well plates (1000 cells/well) and incubated for 24, 48, 72, or 96 h. At each time point, 10 *μ*l of CCK-8 solution was added to each well and the cells were incubated for 2 h at 37°C. The absorbance (450 nm) was measured using a microplate reader (BioTek, Winooski, VT, USA). All of the experiments were repeated at least three times.

### 2.8. Colony Formation Assay

Stable transfected pancreatic cancer cells were trypsinized into a single cell suspension and plated in 6-well plates (approximately 500 cells/well) in triplicate. After incubation for 14 days, cells were gently washed with PBS buffer and fixed with 4% paraformaldehyde and then stained with 0.1% crystal violet. Colonies were photographed and quantified independently. Experiments were repeated three times.

### 2.9. Cell Cycle Assay

Stable transfected pancreatic cancer cells in log phase seeded in 6-well plates were harvested by trypsinization and washed with PBS buffer. For cell cycle analysis, samples were fixed in 70% ethanol, stained with propidium iodide (PI) (Servicebio), supplemented with RNaseA, and analyzed by a flow cytometer (CytoFLEX, Beckman Coulter, Brea, California, USA).

### 2.10. *In Vivo* Tumorigenicity Assay

Balb/c mice were obtained from Shanghai Experimental Animal Center of the Chinese Academy of Sciences (Shanghai, China) and housed under specific pathogen-free conditions. For tumor xenograft assays, stable transfected CaPan-2 (shEI and shCTR) or PANC-1 (EI and SCR) cells were subcutaneously injected into the dorsal flank of 6-week-old nude mice (3 × 10^6^ cells per mouse). The mice were sacrificed 35 days later. Tumors were weighed, and the diameters were measured. The tumor volume was estimated using the formula: (width)^2^ × length/2. The excised tumor tissues were divided into two equal parts and embedded in paraffin or stored at −80°C for further use. All experiments were approved by the Ethical Committee of Ruijin Hospital. All procedures followed the Regulations of Experimental Animal Administration issued by the Ministry of Science and Technology of the People's Republic of China.

### 2.11. Immunofluorescence Staining

Immunofluorescence staining was performed as described previously [14]. Slices were deparaffinized, rehydrated, antigen retrieved, permeabilized, and blocked sequentially. Anti-p62 antibody or anti-Beclin-1 antibody (1 : 200, Servicebio) was incubated with samples at 4°C overnight in a humidified chamber. Fluorescently labeled secondary antibodies were added, and samples were incubated 1 h at room temperature. Finally, nuclei were counterstained with DAPI. The fluorescence images were visualized by a fluorescence microscope (Carl Zeiss, Jena, Germany).

### 2.12. Statistical Analysis

All data in this study are presented as the means ± SEM. Fisher's test or two-tailed Student's *t*-test was performed to determine statistical analyses (GraphPad Prism version 6.0 software). The correlation between EI24 and c-Myc expression in PDAC tissues was analyzed by Pearson correlation analysis. A value of *P* < 0.05 was considered to be statistically significant.

## 3. Results

### 3.1. EI24 Is Downregulated in Pancreatic Cancer Tissues

**Figure 1:**
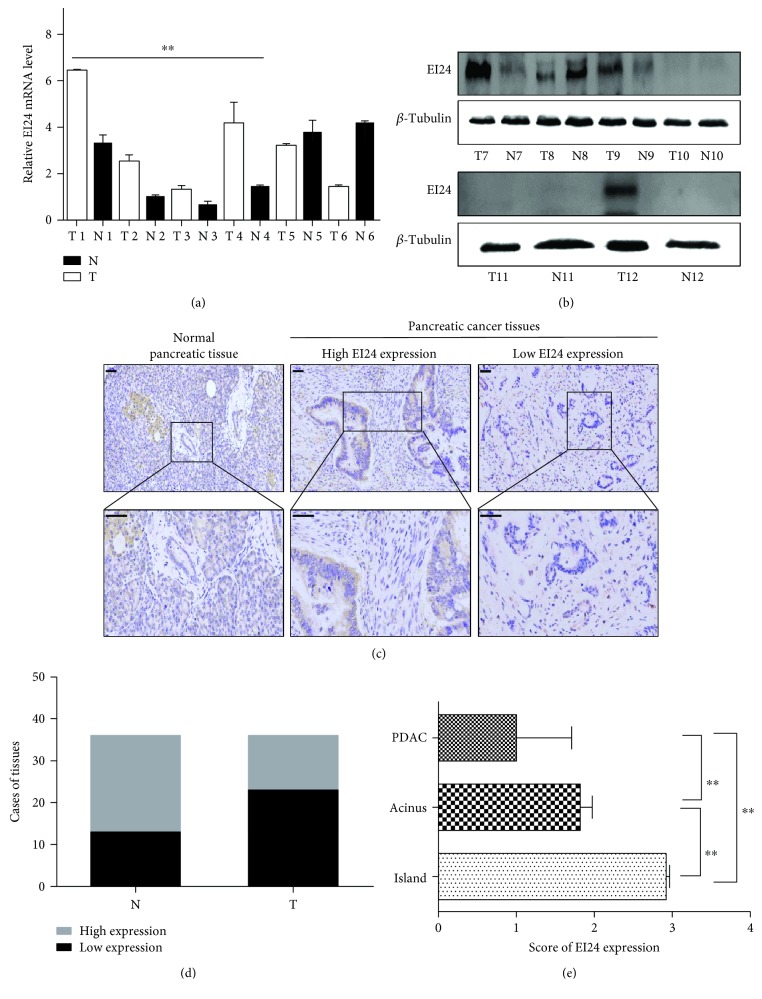
Expression levels of EI24 in PDAC tissues. (a, b) EI24 mRNA and protein expression levels in six pairs of PDAC tissues (T) and adjacent normal tissues (N) detected by RT-qPCR and Western blot analysis, respectively. (c) Immunohistochemical analysis of EI24 expression in pancreatic cancer tissues. Brown color represents positive cytoplasm and nucleus EI24 staining (scale bar represents 50 *μ*m, 200x and 400x magnification). (d) Statistical gram shows that EI24 was downregulated in PDAC tissues (T) than in adjacent normal tissues (N). (e) Statistical analysis showed that EI24 expression was significantly lower in PDAC cells than in normal pancreatic acinus/islands. ^∗^ *P* < 0.05 and ^∗∗^ *P* < 0.01, compared with controls.

We first examined the levels of EI24 mRNA in 6 pairs of tumor tissues and adjacent normal tissues resected from PDAC patients using qRT-PCR analysis (Figure 1(a)). The results identified an elevation of EI24 mRNA levels by approximately 2.24-fold in the tumor tissues compared with the adjacent normal tissues in 4/6 (66.67%) of the samples (*P* < 0.05). However, quantification of levels in all 6 pairs of tissues showed no significant difference compared with controls (Supplementary Figure 1). We next examined the protein level of EI24 in 6 other pairs of resected specimens using Western blot. The results showed elevated or lowered EI24 expression in tumor tissues relative to the adjacent normal tissues (Figure 1(b)).

We next evaluated EI24 levels in tumor tissues from 36 pancreatic cancer patients using immunohistochemistry. EI24 nuclear staining and cytoplasm staining were observed in tumor and normal tissues (Figure 1(c)). Samples were scored as high or low EI24 expression, as described in Materials and Methods. High expression of EI24 was detected in 13 (36.11%) pancreatic cancer cases, while low expression was observed in 23 cases (63.89%) compared with adjacent normal tissues (Table 1 and Figure 1(d)). The low expression rate of EI24 protein was higher in PDAC tissues than that in adjacent normal tissues (*P* = 0.0184). No correlation between EI24 expressions was observed with gender, age, histological differentiation, T or N stage, or metastasis (Table 1). Interestingly, EI24 expression was significantly lower in PDAC tissues than in normal pancreatic acinus/islands (*P* < 0.01) (Figure 1(e)). These data suggested an important role of EI24 in PDAC progression.

### 3.2. EI24 Inhibits Pancreatic Cancer Cell Proliferation *In Vitro*

**Figure 2:**
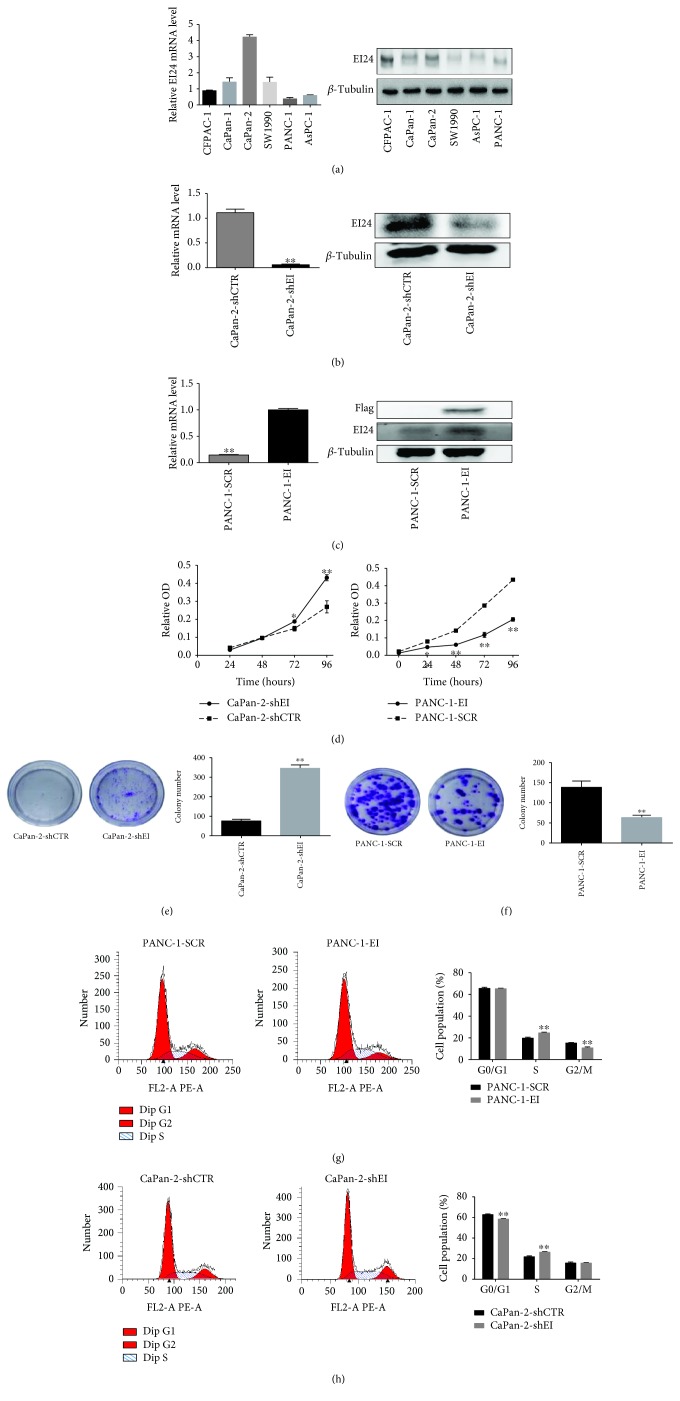
EI24 suppresses pancreatic cancer cell proliferation *in vitro*. (a) EI24 expression in different pancreatic cancer cell lines by qRT-PCR and Western blot analysis. (b, c) CaPan-2 and PANC-1 stable cells with either EI24 knockdown or overexpression, respectively, validated by qRT-PCR and Western blot. (d) CCK8 assays in EI24 knockdown and overexpressing cells. Growth curves of different cells were drawn. (e, f) Stably transfected cells were subjected to colony formation assays. (g, h) Stably transfected CaPan-2 and PANC-1 cells underwent cell cycle analysis by flow cytometry. ^∗^ *P* < 0.05 and ^∗∗^ *P* < 0.01, compared with controls.

We next evaluated EI24 expression in 6 pancreatic cancer cell lines (CFPAC-1, CaPan-1, CaPan-2, SW1990, AsPC-1, and PANC-1). EI24 mRNA was downregulated in the poorly differentiated PDAC cell lines (AsPC-1 and PANC-1) compared with moderately to well-differentiated cells CFPAC-1, CaPan-1, CaPan-2, and SW1990, and Western blot analysis was consistent with these results (Figure 2(a)). These data suggested that EI24 was associated with cancer cell differentiation. CaPan-2 and PANC-1 cell lines were thus chosen to represent cell lines with high and low EI24 expressions, respectively, for subsequent experimental analyses. We next established CaPan-2 and PANC-1 stable cell lines with either EI24 knockdown or overexpression, respectively, as described in Materials and Methods. Stable expressing cell lines, CaPan-2-shEI and PANC-1-EI, and the respective controls were confirmed by qRT-PCR and Western blot analysis (Figures 2(b) and 2(c)).

We next examined the effects of EI24 levels on cellular proliferation using CCK8 assays. Cell growth was remarkably promoted by EI24 depletion in CaPan-2 cells, while EI24 overexpression showed the opposite results (Figure 2(d)). In addition, long-term colony formation assay showed that CaPan-2-shEI cells produced more colonies (77.7%) compared with CaPan-2-shCTR cells (*P* < 0.01), whereas PANC-1-EI cells showed a sharply decreased colony number, by 2.18-fold, compared with PANC-1-SCR cells (*P* < 0.01) (Figures 2(e) and 2(f)). Analysis of cell cycle distribution revealed that PANC-1-EI cells exhibited a 1.43-fold decrease in cells in G2 phase and a 1.25-fold increase in the S phase cells (both *P* < 0.01) (Figure 2(g)). Conversely, knockdown of EI24 resulted in a 1.2-fold increase in the percentage of cells in S phase and a 1.07-fold decrease in cells in G1 phase compared with controls (*P* < 0.01) (Figure 2(h)). Together, these results indicated that EI24 overexpression inhibited pancreatic cancer cell growth, induced cell cycle arrest in S phase, and attenuated cancer cell colony formation.

### 3.3. EI24 Suppresses Pancreatic Cancer Growth *In Vivo*

**Figure 3:**
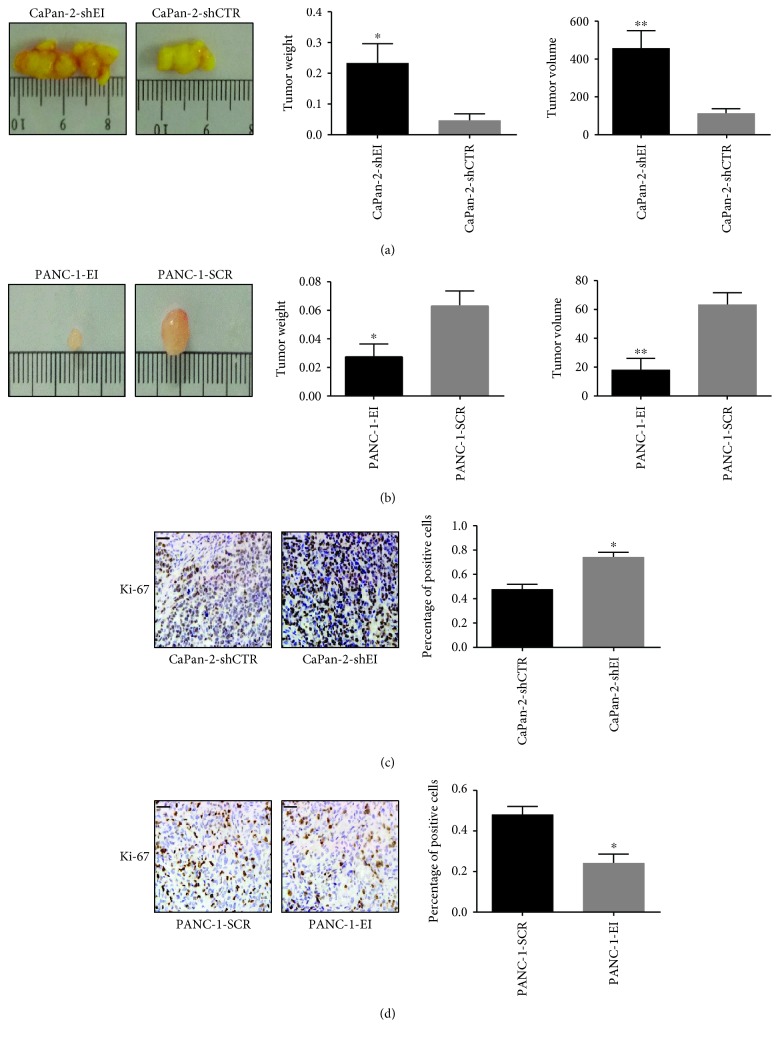
EI24 suppresses pancreatic cancer growth *in vivo*. (a) Representative images of tumors isolated from each group of mice. (b) Statistical gram shows relative changes of tumor weight and volume. (c, d) Immunohistochemical analysis of Ki-67 in tumor tissues (scale bar represents 50 *μ*m, 400x magnification). Percentage of positive Ki-67 staining cells was counted. ^∗^ *P* < 0.05 and ^∗∗^ *P* < 0.01, compared with controls.

To examine the function of EI24 on pancreatic cancer tumorigenesis *in vivo*, we performed tumor formation assays in Balb/c nude mice. Mice subcutaneously implanted with CaPan-2-shEI cells showed a significant increase in tumor weight and volume compared with controls (*P* < 0.05 and *P* < 0.01) (Figures 3(a) and 3(b)). In contrast, the opposite results were shown in mice engrafted with PANC-1-EI cells compared with the control group (*P* < 0.05 and *P* < 0.01) (Figures 3(a) and 3(b)). There were no significant differences in body weight between the experimental groups and controls (data was not shown). These data indicated that EI24 overexpression suppressed pancreatic cancer tumorigenicity *in vivo*, while EI24 knockdown could promote pancreatic cancer tumorigenesis.

Immunohistochemical analysis of the tumors confirmed that Ki-67, a marker of cell proliferation, was marginally increased in tissues isolated from animals treated with CaPan-2-shEI cells compared with the controls (*P* < 0.05) (Figure 3(c)). Likewise, reduced expression of Ki-67 was detected in tumor samples from PANC-1-EI cells (*P* < 0.05) (Figure 3(d)). These data demonstrated that EI24 plays a key role in the proliferation and survival of tumor cells, in line with the *in vitro* results.

### 3.4. EI24 Attenuates Cell Proliferation by Downregulation of c-Myc Involving a Beclin-1/p62/c-Myc Signaling Pathway

**Figure 4:**
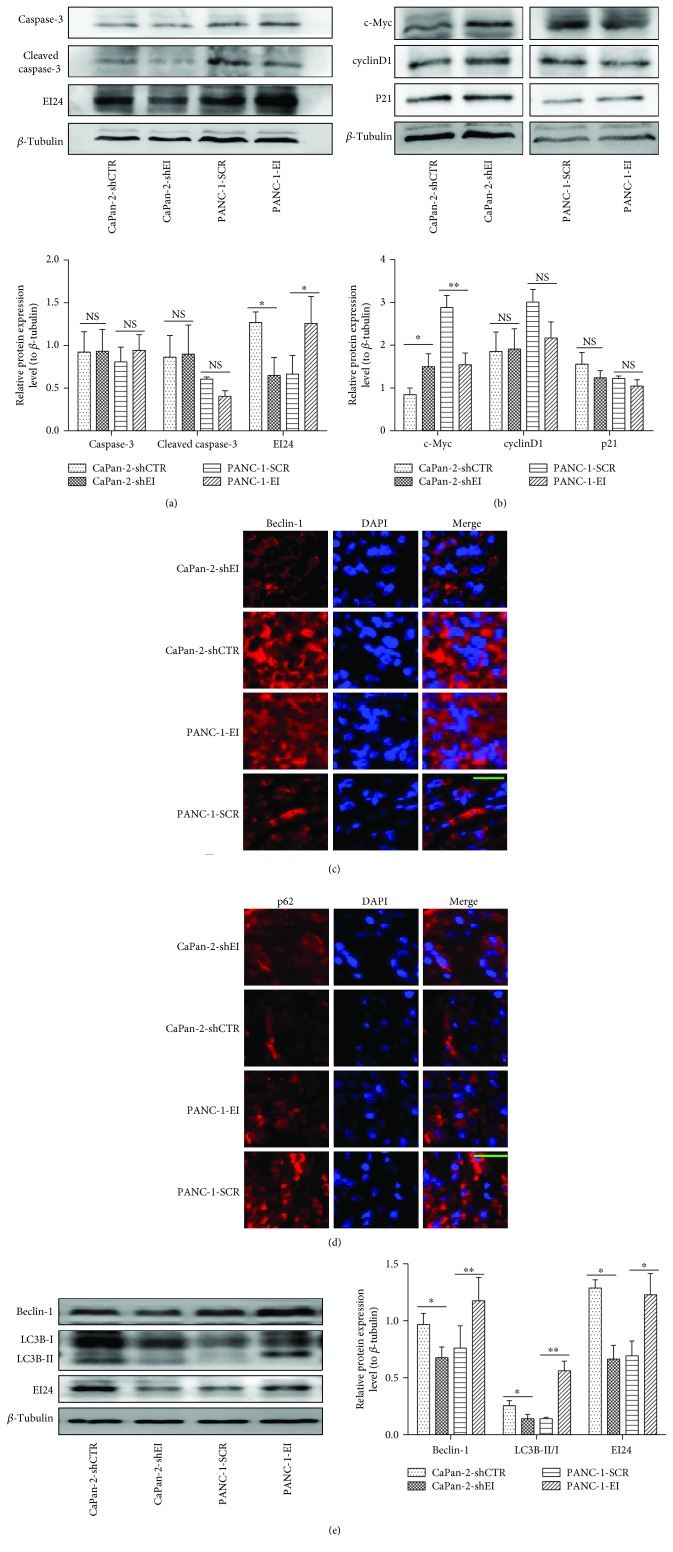
EI24 attenuates cell proliferation by inducing autophagy to regulate the Beclin-1/p62/c-Myc signaling pathway. (a) Expression level of caspase-3 and cleaved caspase-3 was detected by Western blot. (b) Western blot analysis shows cyclinD1, p21, and c-Myc protein levels in cancer cells. (c, d) Immunofluorescence analysis of Beclin-1 and p62 expression in xenograft tumor tissues. EI24 increased merged fluorescence image of Beclin-1 (red, Cy3 stained) with the nucleus (blue, DAPI staining) was observed, accompanied with a decrease of p62. Photos were telegraphed with 400x magnification. Scale bar represents 50 *μ*m. (e) Expression levels of EI24-induced autophagy-related protein LC3B and Beclin-1 were detected by Western blot. ^∗^ *P* < 0.05, ^∗∗^ *P* < 0.01, and NS *P* > 0.05, compared with controls.

Cleaved caspase-3 is an activated form of caspase-3 and functions as a marker of apoptosis. To examine whether the antitumor activity of EI24 involved induction of apoptosis, we examined caspase-3 expression levels in the stable cell lines with overexpression or downregulation of EI24 by Western blotting, but no apparent differences in protein levels were detected (Figure 4(a)). These results indicate that caspase-dependent apoptosis is not the mechanism by which EI24 affects cell viability. We also examined the effect of EI24 on the expression of several cell cycle regulators, including cyclinD1, p21, and c-Myc, in the stable pancreatic cancer cell lines using Western blotting assay (Figure 4(b)). Neither cyclinD1 nor p21 showed changes in expression in either the EI24 overexpressing or downregulated cell lines compared with controls. However, we observed a marked decline in the expression level of c-Myc in PANC-1-EI cells compared with controls, while an increase was observed in CaPan-2-shEI cells compared with controls. These results indicate that the overexpression of EI24 caused decreased c-Myc levels, resulting in growth suppression in pancreatic cancer cells. To validate the correlation between EI24 and c-Myc expression, we also examined the c-Myc level in human pancreatic tissues. Correlation studies showed that a high EI24 level correlated with low c-Myc expression in PDAC specimens. Conversely, a low EI24 level correlated with high c-Myc expression (Supplementary Figures 2A and B). Taken together, these results suggest that the absence of EI24 inversely correlates with c-Myc upregulation in PDAC and EI24 may exert its antitumor function partially through inhibiting c-Myc oncogenic function.

A previous study found that Beclin-1 accelerates the degradation of c-Myc and that the Beclin-1/p62/c-Myc signaling pathway functions in regulating tumor proliferation [11]. EI24 is an autophagy-associated transmembrane protein and an essential component of basal autophagy in mammals [12]. We wondered if the Beclin-1/p62/c-Myc signaling pathway is activated and functions in pancreatic cancer and whether EI24 plays a role in this pathway. To first clarify whether the suppression of pancreatic cancer tumorigenicity induced by EI24 was associated with autophagy, we examined Beclin-1, which is essential for autophagy upregulation, by immunofluorescence analysis in xenograft tissues. As shown in Figure 4(c), the expression of Beclin-1 increased by overexpression of EI24, while EI24 knockdown showed reduced levels compared with controls. Examination of the clearance of p62 (Figure 4(d)), another distinct feature of autophagy, was consistent with these results. Based on these findings, we concluded that EI24 overexpression in PDAC activates autophagy, and we speculated that this may be associated with the effects of EI24 on tumorigenesis *in vivo*. We next considered whether the autophagy induced by EI24 correlated with the deregulation of c-Myc. Overexpression of EI24 promoted conversion of LC3B-I to LC3B-II, and the opposite results were shown in cells with the knockdown of EI24 (Figure 4(e)). Ectopic expression of EI24 also caused induction of Beclin-1 (Figure 4(e)), and this paralleled the reduction of c-Myc. Together, these data suggested that EI24 suppresses tumor proliferation possibly by the activation of autophagy to regulate the Beclin-1/p62/c-Myc signaling pathway.

## 4. Discussion

EI24 has been previously linked with inhibiting cancer cell growth and proapoptotic functions [7]. In the present study, we demonstrated decreased expression of EI24 in human PDAC compared with adjacent normal tissues and found higher EI24 expression in moderately to well-differentiated PDAC cells compared with poorly differentiated cells. We further clarified that EI24 acts as a tumor suppressor in PDAC cell lines by inhibiting cell proliferation, decreasing colony formation, and inducing cell cycle arrest. Furthermore, these effects were independent of caspase-dependent apoptosis. Because the induction of cell differentiation requires cell growth inhibition or cell cycle arrest [15], our results may give a reasonable explanation for the differential expressions of EI24 in differentiated cells. Notably, these findings could link the EI24 low expression level in PDAC with its tumor suppressor role. Downregulation of EI24 may result in a deregulated tumor suppression system and promoted tumorigenesis. Alternatively, EI24 was downregulated in PDAC than normal pancreatic acinus/islands. Considering acinar-to-ductal metaplasia in PDAC initiation [16], deficiency of EI24 may potentiate the initiation of tumors. Further studies will be conducted in pancreatic intraepithelial neoplasia (PanIN) cells and tissues to explore the effects of EI24 on tumor initiation.

c-Myc plays an important role in regulating cell proliferation, differentiation, and apoptosis [4]. Li et al. revealed that the PI3K/AKT signaling pathway was involved in PSCA-promoted cell proliferation and cell cycle progression by upregulating c-Myc in prostate cancer [17]. Moreover, the previous work demonstrated that c-Myc functions in pancreatic cancer as a central oncogene [4]. Our study found that the ectopic expression of EI24 reduced cell proliferation, impaired cell cycle progression, and reduced tumorigenesis. Furthermore, EI24 knockdown accelerated cell cycle transition from G1 phase to S phase, suggesting a promotion of cell proliferation in the absence of EI24; in contrast, EI24 overexpressing cells showed an increase in S phase cells accompanied with a decrease in G2 phase cells, indicating an S phase arrest. The Ki67 immunohistochemistry staining results were consistent with these data. To examine the mechanisms underlying EI24-mediated tumor suppression, we examined protein levels of several critical cell cycle and growth regulators and confirmed alterations in c-Myc in response to EI24. IHC staining of human pancreatic tissues also showed the inverse correlations between EI24 and c-Myc. The recent study confirmed that the activation of autophagy promotes the degradation of c-Myc, leading to reduced cell proliferation [11]. Thus, a similar degradation mechanism may occur in pancreatic cancer, in which the downregulation of c-Myc occurs in response to reinforced EI24 expression. Consistent with the demonstrated role for EI24 in autophagy, Beclin-1/p62 expression levels were altered in response to EI24, along with changes in c-Myc levels. Together, these findings reveal a possible EI24/Beclin-1/p62/c-Myc signal pathway. However, further studies are required to elucidate the mechanism underlying this pathway.

Previous studies demonstrated a function for EI24 in the ubiquitin-proteasome pathway-autophagy crosstalk [13], which further supports its potential link to c-Myc. Other studies have proven that c-Myc correlated with preacinar to acinar maturation and pancreatic homeostasis [18], providing insight into differentiation and carcinogenesis. In view of evidence that the induction of a dedifferentiated state caused by c-Myc overexpression contributes to pancreatic carcinogenesis [18] and our findings, this may help explain the downregulation of EI24 in PDAC compared with normal pancreatic acinus/islands and lower expression level in poorly differentiated cancer cells compared with well- and moderately differentiated cancer cells. This indicates that c-Myc is an important target of EI24 and essential to its role as a tumor suppressor. More studies are required to verify the functional interaction between EI24 and c-Myc and to investigate other molecular targets and mechanisms for EI24 to clarify its role in tumor initiation and progression.

Autophagy is required for maintaining cellular homeostasis and has been linked with both protumorigenic and tumor suppression functions [10]. The role of autophagy in PDAC is unclear, and the exact mechanisms have yet to be elucidated. Our study demonstrated that enhanced expression of EI24 activates autophagy *in vitro* and *in vivo* in pancreatic cancer cells. Our results suggest that the overexpression of EI24 affected tumorigenesis in pancreatic cancer by inducing autophagy, resulting in the downregulation of c-Myc and suppression of proliferation, as well as causing cell cycle arrest. Notably, in contrast with other findings, EI24 had no impact on apoptosis in the current study. Researchers have demonstrated autophagy-related cell death as an anticancer mechanism in PDAC [19], and induction of autophagy has become an emerging strategy for PDAC treatment [20]. Thus, autophagy may affect tumor progression by contributing to cell death independent of apoptosis. We plan to further examine the relation between EI24-induced autophagy and cell death and the mechanism underlying the crosstalk between EI24-mediated autophagy and apoptosis in pancreatic cancer in future studies.

In conclusion, our results not only indicate that EI24 is downregulated in PDAC tissues compared to the adjacent normal tissues but also provide evidence that EI24 exhibits a potent tumor suppressor effect on pancreatic cancer cells by inhibiting cell proliferation both *in vitro* and *in vivo* and inducing cell cycle arrest at S phase. The possible mechanism for this effect might involve EI24-mediated autophagy-related degradation of c-Myc, through regulating Beclin-1 and p62. Our present study provides insight into the crosstalk between autophagy and cell proliferation involving a possible EI24/Beclin-1/p62/c-Myc signaling pathway. These findings suggested that EI24, as a potential tumor suppressor, might serve as an effective therapeutic target against pancreatic cancer.

## Figures and Tables

**Table 1:** Associations of EI24 expression with clinicopathological characteristics in PDAC patients.

Clinical parameters	Total (36)	EI24 expression		*P* value
		Low (*n* = 23)	High (*n* = 13)	
Gender				0.2651
Male	21	15	6	
Female	15	8	7	
Age (years)				0.6359
≤60	10	7	3	
>60	26	16	10	
Histological differentiation				0.9685
Moderate/~well	22	14	8	
Poor/~moderate	14	9	5	
T classification				0.6436
T1 + T2	7	5	2	
T3 + T4	29	18	11	
N classification				0.3187
Absent	21	12	9	
Present	15	11	4	
Metastasis				0.6789
No	29	19	10	
Yes	7	4	3	

## Data Availability

The research data used to support the findings of this study are available from the corresponding authors upon request.

## Supplementary Materials

Supplementary Materials Supplementary Table 1: primer sequences of RT-qPCR (5′ to 3′) test. Supplementary Table 2: primary/secondary antibodies for Western blot analysis. Supplementary Figure 1: EI24 mRNA of PDAC patients has no significant difference. Supplementary Figure 2: expression of c-Myc inversely correlates with EI24 expression.
