# Supplementary material for: EI24 Suppresses Tumorigenesis in Pancreatic Cancer via Regulating c-Myc
Source: Gastroenterol Res Pract. 2018 Oct 2;2018:2626545. doi: 10.1155/2018/2626545 (PMC6189671; doi:10.1155/2018/2626545)
Supplement: Supplementary Materials — Supplementary Table 1: primer sequences of RT-qPCR (5′ to 3′) test. Supplementary Table 2: primary/secondary antibodies for Western blot analysis. Supplementary Figure 1: EI24 mRNA of PDAC patients has no significant difference. Supplementary Figure 2: expression of c-Myc inversely correlates with EI24 expression. [file 2626545.f1.pdf]

Supplementary Table 1. Primer sequences of RT-qPCR (5' to 3') test.

Gene	Sequences
hEI24	Forward: GCAAGTAGTGTCTTGGCACAGAG<br>Reverse: AGAACACTCCACCATTCCAAGC
hβ-actin	Forward: AGCACAGAGCCTCGCCTTT<br>Reverse: GTTGTCGACGACGAGCG

Supplementary Table 2. Primary/ secondary antibodies for western blot analysis.

Primary/ secondary antibodies	Corporation	Dilution ratio
Rabbit anti- EI24	Sigma-Aldrich	1:500
Rabbit anti-Caspase-3	Cell Signaling Technology	1:1000
Rabbit anti- cyclinD1	Cell Signaling Technology	1:1000
Rabbit anti- p21	Cell Signaling Technology	1:1000
Rabbit anti-c-Myc	Cell Signaling Technology	1:1000
Rabbit anti- LC3B	Cell Signaling Technology	1:1000
Rabbit anti- Beclin-1	Cell Signaling Technology	1:1000
Rabbit anti-β-tubulin	Cell Signaling Technology	1:1000
Mouse anti-DDDDK-Tag	ABclonal	1:2000
HRP-conjugated anti-rabbit IgG	Cell Signaling Technology	1:5000
HRP-conjugated anti-mouse IgG	Cell Signaling Technology	1:5000

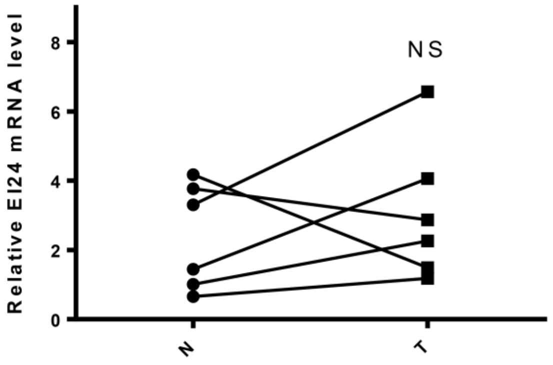

Supplementary Figure 1. EI24 mRNA of PDAC patients has no significant difference.

EI24 mRNA expression levels in six pairs of PDAC tissues (T) and adjacent normal tissues (N) detected by RT-qPCR. β-actin mRNA expression was used as an internal control. NS means P > 0.05 compared with controls.

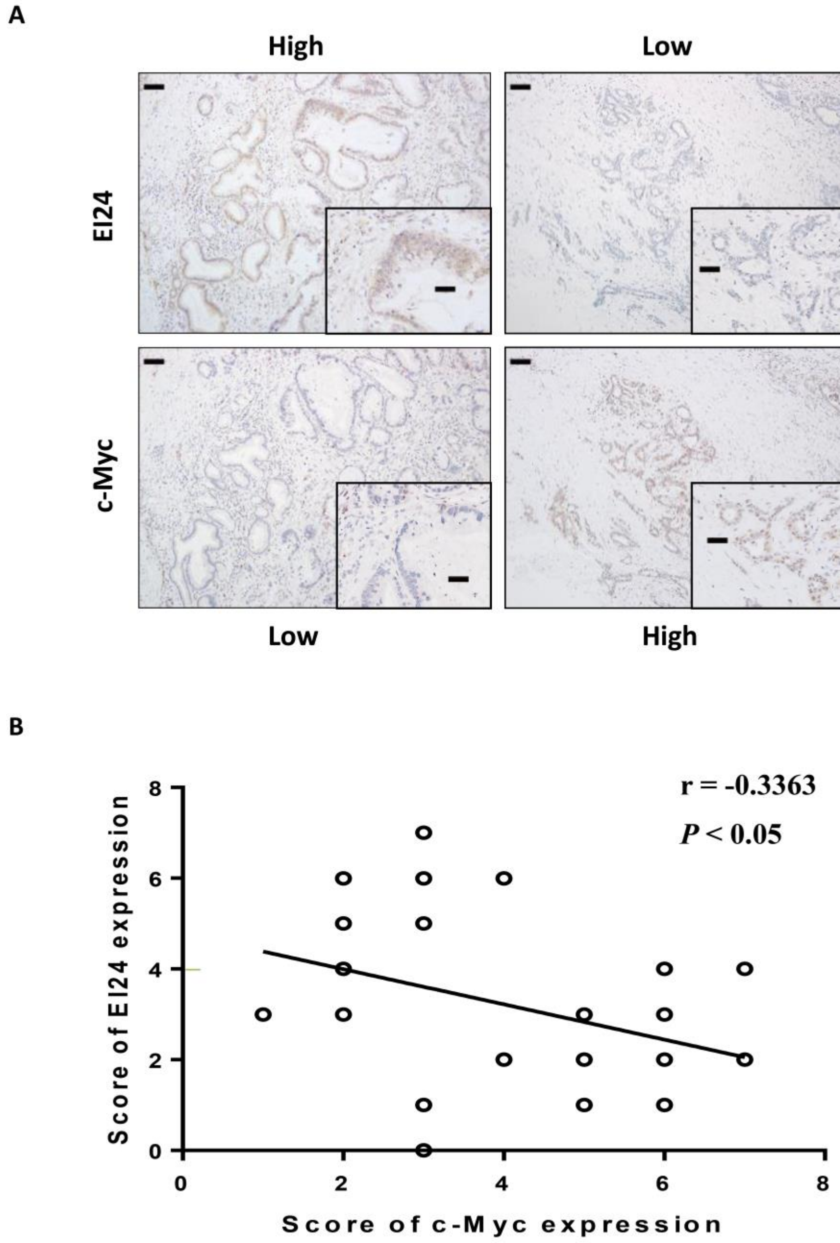

Supplementary Figure 2. Expression of c-Myc inversely correlates with EI24 expression.

A. Representative IHC staining of EI24 and c-Myc in human pancreatic adenocarcinoma tissue (scale bar, 100 μm, inset scale bar, 50 μm; 100× and 200× magnification). **B**. The correlation between EI24 and c-Myc expression in PDAC tissues was analyzed by Pearson correlation analysis ($P < 0.05$, r = −0.3363).
